# The Influence of Living Near Roadways on Spirometry and Exhaled Nitric Oxide in Elementary Schoolchildren

**DOI:** 10.1289/ehp.10943

**Published:** 2008-08-01

**Authors:** Robert Dales, Amanda Wheeler, Mamun Mahmud, Anna Maria Frescura, Marc Smith-Doiron, Elizabeth Nethery, Ling Liu

**Affiliations:** 1 Faculty of Medicine, University of Ottawa, Ottawa Hospital Research Institute, Ottawa, Ontario, Canada; 2 Health Canada, Ottawa, Ontario, Canada; 3 Air Health Science Division, Water, Air, and Climate Change Bureau, Ottawa, Ontario, Canada; 4 Biostatistics and Epidemiology Division, Environmental Health Science and Research Bureau, Health Canada, Ottawa, Ontario, Canada; 5 Immunization and Respiratory Infections Division, Public Health Agency of Canada, Ottawa, Ontario, Canada

**Keywords:** air pollution, environment, nitric oxide, respiratory system, spirometry

## Abstract

**Background:**

Living near major roadways has been associated with an increase in respiratory symptoms, but little is known about how this relates to airway inflammation.

**Objective:**

We assessed the effects of living near local residential roadways based on objective indicators of ventilatory function and airway inflammation.

**Methods:**

We estimated ambient air pollution, resolved to the level of the child’s neighborhood, using a land-use regression model for children 9–11 years of age. We also summed the length of roadways found within a 200-m radius of each child’s neighborhood. We had measurements of both air pollution exposure and spirometry for 2,328 children, and also had measurements of exhaled nitric oxide (eNO) for 1,613 of these children.

**Results:**

Each kilometer of local roadway within a 200-m radius of the home was associated with a 6.8% increase in eNO (*p* = 0.045). Each kilometer of any type of roadway (local, major, highway) was also associated with an increase in eNO of 10.1% (*p* = 0.002). Each microgram per cubic meter increase in PM_2.5_ was associated with a 3.9% increase in eNO (*p* = 0.058) and 0.70% decrease in forced vital capacity (FVC) expressed as a percentage of predicted (*p* = 0.39). Associations between roadway density and both forced expired volume in 1 sec and FVC were negative but not statistically significant at *p* < 0.05.

**Conclusion:**

Traffic from local neighborhood roadways may cause airway inflammation as indicated by eNO. This may be a more sensitive indicator of adverse air pollution effects than traditional measures of ventilatory function.

Acute and chronic exposure to urban air pollution in North America and Europe has been associated with increased respiratory symptoms, hospitalizations, and death from respiratory diseases (Bascom et al. 1996; Brunekreef and Holgate 2002; Dockery and Pope 2002; Stieb et al. 2002). Exposure to traffic-related air pollutants, often indicated by ambient nitrogen dioxide or proximity to traffic, has been associated with increased reports of wheeze and asthma in some, but not all, studies (Heinrich and Wichmann 2004; Janssen et al. 2003; Kim et al. 2004; Lin et al. 2002). We compared two different methods of estimating the children’s exposure to traffic-related air pollution. We measured the density of local roadways surrounding the child’s home, and we also used land-use regression (LUR) model results to estimate each child’s exposure to individual traffic-related pollutants at the postal code level, an area equivalent to about 30 homes or one large apartment building. This latter technique has been shown to improve the spatial discrimination in air pollution concentrations compared with using central site monitoring data alone (Pouliou et al. 2008).

Ventilatory function, which is commonly measured by forced expired volume in 1 sec (FEV_1_) and forced vital capacity (FVC), reflects lung structure, airway caliber, and lung size. In addition, we measured exhaled nitric oxide (eNO), a measure of airway inflammation in asthma.

Inflammatory cytokines induce the enzyme nitric oxide synthetase type II, which then augments the production of eNO by endothelial, epithelial, and inflammatory cells in the airways (American Thoracic Society 2005; Payne 2003; Wark and Gibson 2003). There have been a few studies of eNO and air pollutants in children but, to our knowledge, only one in which road density was the exposure of interest (Delfino et al. 2006; Fischer et al. 2002; Holguin et al. 2007; Koenig et al. 2003; Steerenberg et al. 2001).

Airway inflammation is associated with and may be a precursor of structural airway changes. This raises the question of whether eNO may be a more sensitive marker of an adverse pulmonary effect of air pollution than traditional measures of ventilatory function. The present study allowed us to compare results based on two different measures of exposure, LUR-predicted air pollution concentrations and roadway density at the postal code level, and two different measures of a possible physiologic response, ventilatory function and airway inflammation.

## Materials and Methods

### Study design and recruitment

We used a cross-sectional prevalence design. We distributed questionnaires through the Windsor, Ontario, school system to students in grades 4–6, which they carried home in their backpacks. The child’s parent or guardian was asked to complete the questionnaire and return it to the school along with informed consent for the child to participate in the two different lung function tests that we conducted at the schools. This study was approved by the Research Ethics Board of Health Canada.

### Air pollutant exposure estimates

Using methods previously developed for LUR models, we similarly estimated air pollution concentrations for each child’s residence at the postal code level (Wheeler et al. 2008). To develop the model, we undertook air pollution monitoring at 50 sites for a 2-week period, during four seasons, to provide an estimate of the average annual concentrations of traffic-related air pollutants. Because measurements from each of the four monitoring seasons were highly correlated with the estimated annual average, we used this site-specific average as the dependent variable in the LUR model. Measured pollutants included sulfur dioxide, NO_2_, coarse particulate matter [2.5–10 μm in aerodynamic diameter (PM_2.5–10_)], fine PM (PM_2.5_), and PM_2.5_ black smoke (Demokritou et al. 2001; International Organization for Standardization 1993; Lee et al. 2006).

Using ArcGIS 9.0 (ArcMap 9.0; ESRI, Redlands, CA, USA), we created LUR models using road network data, population and dwelling counts, and any Detroit- or Windsor-based industrial point sources (Briggs et al. 1997; Jerrett et al. 2005a, 2005b; Wheeler et al. 2008) The final NO_2_ LUR model included distance to the Ambassador Bridge, highways, and population density, and the PM_2.5_ model included major roads, highways, and local roads. As a secondary analysis, we also estimated exposure to air pollutants in the postal code of the child’s school using the LUR, and then calculated a time-weighted exposure combining home and school exposures for the child. To separate out the health effects associated with long-term exposures to air pollution, which we estimated using the LUR, we also calculated the short-term exposures to air pollution, using the 24-hr daily mean preceding the lung function measurements, from the two National Air Pollution Surveillance Network (NAPS) monitoring sites in Windsor (NAPS 2008).

### Measured proximity to roadways

This method of estimating exposure to traffic has been previously reported (Gilbert et al. 2005; Jerrett et al. 2007; McConnell et al. 2006). We determined the location of the child’s home by the six-digit postal code, which can resolve the location to a group of homes on one side of a street (an average of 30 homes) or an apartment building. We calculated the total length of roadways within a 200-m radius around the home (based on postal code) using GIS software and information from CanMap Major Roads and Highways software, developed by DMTI Spatial Inc. (Markham, ON, Canada). It provides a standardized “cartographic road classification” of Canadian roadways: expressway, primary highway, secondary highway, major roadways, and local roadways. We also calculated the simple linear distances between the Ambassador Bridge, a major truck transportation route between Canada and the United States, and the child’s neighborhood.

### Questionnaire

For all subjects, a questionnaire inquired about place of residence, postal code, respiratory infection within the preceding 2 weeks, asthma medications in the preceding week, number of cigarettes smoked in the home, and presence of indoor pets. Within the preceding 6 months, most subjects had participated in a previous questionnaire study that provided extensive information on usual respiratory symptoms and their residential environment. We created a database by electronically scanning the questionnaires, which we verified by 100% manual data verification.

### Spirometry

Spirometry was performed once for each child at the school by certified respiratory therapists using American Thoracic Society criteria (American Thoracic Society 1995). KoKo Spirometers (Pulmonary Data Services, Inc., Louiseville, CO, USA) were calibrated daily and results adjusted for temperature, barometric pressure, age, height, and sex according to Polgar and Promadhat (1971). A maximum of eight FVC maneuvers were carried out in an attempt to achieve three acceptable flow-volume loops, with two being within 200 mL for FVC and FEV_1_. The value assigned to the participant was the largest acceptable value within 200 mL of a second value.

### eNO

Before spirometry, single-breath online measures of eNO were performed using an Eco Physics CLD AL MED chemiluminescence analyzer (Eco Medics AG, Duernten, Switzerland) and SpiroWare 88 software (Eco Medics AG). The equipment specifications (e.g., sensitivity < 1 ppb nitric oxide) and test performance met American Thoracic Society and European Respiratory Society Guidelines (American Thoracic Society 2005). Before performing a slow vital capacity maneuver over at least 6 sec at 0.05 L/sec, subjects took three tidal volume breaths through a DENOX 88 (Eco Medics AG) containing an NO-absorbing cartridge that scrubbed the ambient air to 0–1.5 ppb NO. The test was repeated a maximum of eight times in an attempt to obtain at least two acceptable plateau eNO values within 10%. The value assigned to the participant was the mean of these two values.

### Statistical analysis

We used multivariate linear regression to test the association between the different exposure estimates and percent predicted FEV_1_ and FVC. These we adjusted for ethnic background (Caucasian vs. other), smokers at home, pets at home, acute respiratory illness (cold/bronchitis/pneumonia) in the preceding 2 weeks, and any medication for wheezing/asthma taken in the preceding 2 weeks. For the associations between exposure and eNO, we adjusted for the afore-mentioned covariates and also for the children’s height, weight, age, and sex. We log-transformed the eNO model before analysis to normalize the residuals. To remove any unwanted seasonal variability in each of our models, we also included a variable to represent each month during the study from February through June. To control for any acute effects of air pollution on lung physiology, we included a variable representing the NAPS estimate of the previous 24-hr daily average. We selected confounders based on their relation to exposure and health outcomes; we also evaluated interactions of selected covariates with exposure.

We performed univariate and stratified analyses to investigate any potential interactions and confounding. Stepwise regression analyses considered all main effects and first-order interaction products. We inspected the Wald chi-square statistic and *p*-value for each first-order interaction product. If the *p*-value was statistically significant (*p* < 0.10), we retained the main effect and interaction product. We then ran the final model using only the selected variables (both main and interactions), and we retained covariates only if they were significant at *p* < 0.05 or if they confounded the exposure–outcome relationship (i.e., a change of 10% in the coefficient for exposure). We completed all data management and regression modeling in SAS, version 9.1 (SAS Institute Inc., Cary, NC, USA).

## Results

A total of 2,402 children consented to participate and came for testing, and of these, 2,328 had air pollution measures assigned to them and had acceptable and reproducible spirometry data. A total of 1,788 had acceptable and reproducible eNO, and of these, 1,613 also had air pollution measures available.

The mean age of the children was 11 years; approximately half were male, and three-quarters were Caucasian (Table 1). Just over half had pets at home, and < 0.2% reported any smoking exposures. Exposures to air pollutants and roadways were similar between participants grouped by the presence or absence of these specific characteristics. In the present study, the 24-hr median for PM_2.5_ was 15.4 μg/m^3^ using the LUR estimates (Table 2). There was little variation in PM_2.5_ across the city, with a 5th percentile of 14.2 μg/m^3^ and a 95th percentile of 17.2 μg/m^3^. Only 5% of the children lived within 1.7 km the Ambassador Bridge. FEV_1_ and FVC, when unadjusted for height, age, and sex, were similar across tertiles of air pollutants and roadways (Table 3). Tertiles did not have equal numbers of children in each because of many “ties”—for example, length of all roadways, resolved to two decimal places, had 116 identical values at the cut point of 2.0 km. The FEV_1_ and FVC were approximately 40 mL less in the highest compared with the lowest tertiles of NO_2_, SO_2_, and coarse PM. eNO was 8% greater in the highest tertile compared with the lowest tertile of local roadway density. None of these differences was statistically significant at the *p* < 0.05 when tested by analysis of variance.

When the air pollution estimates were expressed as continuous measures, no adjusted associations were statistically significant with percent predicted FEV_1_ or FVC (Table 4). The natural logarithm of eNO was associated with PM_2.5_ at a significance level of *p* = 0.058, equivalent to a 3.9% [95% confidence interval (CI), −0.11 to 7.84] increase in eNO per μg/m^3^ increase in PM_2.5_. NO_2_, SO_2_, black smoke, and coarse PM showed positive but nonsignificant associations with eNO.

Length of all roadways within a 200-m buffer near the home was positively and significantly associated with eNO (β = 0.0964; 95% CI, 0.034–0.158; *p* = 0.002). For every one-unit (1-km) increase in combined length of roadways within a 200-m radius of the neighborhood, there was an associated 10.1% (exponent 0.096) change in eNO, with all other variables in the model being held constant. Interestingly, we also saw a positive effect when we considered only local roadways near the home and excluded major roadways and highways, with a regression estimate of 0.065 (95% CI, 0.0015–0.129; *p* = 0.045). Each 1-km increase in local roadways was associated with a 6.8% increase in eNO, with all other variables in the model held constant.

### Subgroup analyses

To determine whether the acute effects of air pollution were influencing the observed results, we used data from the two NAPS monitors in Windsor to adjust for the mean air pollution concentrations 24 hr and 48 hr before the physiologic testing. This caused no significant changes to the results, suggesting that the acute exposures did not account for the observed effects upon pulmonary function and that these outcomes were related to the annual averaged exposure and were hence more likely to be chronic effects. Also, accounting for exposure at school did not influence the observed air pollution health effect estimates that we based on home exposures alone. We dichotomized the data by several variables to look for effect modification: adult respondent with some versus no postsecondary education; total household income greater than versus less than Can$35,000; and family had moved within the previous 4 years versus had not moved. Values for 95% CIs for the air pollution–lung function association overlapped between each stratum. When we limited analysis to those children with both eNO and spirometry measures available, the association between eNO and roadway density, but not the association between spirometry and roadway density, remained statistically significant. Whether or not there was a history of physician-diagnosed asthma, eNO was positively associated with roadway density and PM_2.5_, but it was statistically significant only for the former exposure metric (Table 5).

## Discussion

Among elementary-school age children, we found that the concentration of eNO, a measure of asthma-related airway inflammation (American Thoracic Society 2005), was positively and significantly related to increased roadway density within a 200-m buffer around the neighborhood. We saw effects for all types of roadways combined and also for local residential roadways alone. eNO concentrations were not significantly associated with LUR-estimated NO_2_, SO_2_, black smoke, coarse PM, or PM_2.5_. Ventilatory lung function was not significantly associated with roadway density or air pollutant concentrations.

### Respiratory health effects related to roadways

Previous studies have found adverse effects on children’s respiratory health from living near major roadways or freeways. McConnell et al. (2006) recently reported that living within 75 m of a major road was associated with an elevated risk of reporting lifetime asthma with an odds ratio of 1.29 (95% CI, 1.01–1.86). Ryan et al. (2005) reported increased wheezing among infants living within 100 m of stop-and-go traffic. Compared with those living at least 1,500 m away from a freeway, children living within 500 m had an 81 mL (95% CI, 18.0–143.0) greater decrease in FEV_1_ over an 8-year period (Gauderman et al. 2007). In the present study we found that even local neighborhood roadways may also have adverse respiratory effects.

The influence of air pollution on eNO in children has been the subject of a few studies (Delfino et al. 2006; Fischer et al. 2002; Koenig et al. 2003; Steerenberg et al. 2001), but apart from the present study, we could find only one (Holguin et al. 2007) that used road density, a more direct measure of traffic exposure. Holguin et al. (2007) reported that among 95 children with physician-diagnosed asthma, an interquartile increase in road density within a 200-m “home buffer” was associated with a 17% (95% CI, −2 to 40; *p* = 0.09) increase in eNO. Buffers of 50 and 100 m achieved higher levels of statistical significance (*p* = 0.03 and *p* = 0.005, respectively). Holguin et al. (2007) found no significant effects in the combined study group (95 with asthma and 99 without diagnosed asthma), whereas we found effects in both those with and without a history of physician-diagnosed asthma, perhaps partly because of a larger sample size. In the study by Holguin et al. (2007), the individually measured traffic-related pollutants NO_2_, PM_2.5_, elemental carbon, and traffic counts were not related to eNO. The present study confirms the eNO–roadway density association reported by Holguin et al. (2007). These two studies suggest that roadway density may be a better indicator of traffic-related respiratory health effects than are individual air pollutant measures. This association persisted in our study after adjustment for air pollutant levels (NO_2_, SO_2_, PM_2.5_) within the previous 24 and 48 hr of the eNO measure, indicating that it was not confounded by an unmeasured acute effect.

### The effect of air pollution on eNO

This observed association between roadway proximity and eNO is relatively unique but is consistent with the results of studies using measured air pollutants. In a panel study of 45 schoolchildren with asthma carried out in Southern California by Delfino et al. (2006), eNO was greater on days of higher concentrations of personal PM_2.5_, elemental carbon, and NO_2_. A 24-μg/m^3^ increase in personal PM_2.5_ was associated with a 1.1-ppb (95% CI, 0.1–1.9) increase in eNO. Among 19 children with asthma in Seattle, Mar et al. (2005) reported a 6.9-ppb (95% CI, 3.4–10.6) increase in eNO lagged by 1 hr for a 10-μg/m^3^ increase in PM_2.5_. Steerenberg et al. (2001) found an association between PM_10_ and eNO in children not selected for asthma in urban but not rural areas of the Netherlands. Fischer et al. (2002) reported increased eNO but no significant spirometry changes related to measured air pollutants in unselected children and suggested that eNO may be a more sensitive marker, similar to the observations of the present study. Heinrich and Wichmann (2004) reviewed the published effects of traffic-related air pollution on asthma and allergic disease and concluded that the epidemiologic evidence was “conflicting.” Reviewed studies included symptoms and occasionally measured allergic sensitization, but eNO was not considered. Increases in eNO in those children with a higher density of roadways near their home would not be inconsistent with an allergic mechanism. Increased eNO is a useful clinical marker of asthma, especially atopic asthma, and has been associated with eczema, hay fever, and elevated levels of immunoglobulin E and eosinophils (American Thoracic Society 2005; Brussee et al. 2005; Payne 2003).

### Exposure estimates

Modeling exposure to air pollutants provides a better estimate of exposure to traffic-related pollutants than do self-reported estimates (Heinrich et al. 2005). LUR modeling of air pollution concentrations has been shown to capture more spatial variability within an urban center than using only concentrations measured at the closest available central site monitor (Pouliou et al. 2008). Jerrett et al. (2005b) reported that within-city gradients in exposure to PM_2.5_ may be larger than between-city variability in exposure, and found that LUR provided 50–90% greater mortality estimates. Ryan et al. (2005) found a significant association between infant wheezing and LUR estimates of diesel PM concentrations between 0.3 and 0.9 μg/m^3^. Exposure was more reliably estimated using LUR than simply distance from roadways.

Although better than using only data from fixed-site central monitors, the LUR estimates for Windsor did not have a large degree of variability. Proximity to the Ambassador Bridge was the primary predictor of NO_2_ in the model, but NO_2_ drops off within 200 m of its point source, and very few people lived within 200 m of the bridge. The closest third of our study participants lived an average of 5.8 km from the bridge. Gilbert et al. (2003) demonstrated that background levels of NO_2_, a typical indicator of traffic-related pollutants, are reached by about 200 m from the roadway, suggesting that any potential influence of this source would be undetected for this population.

Local roadways contributed little to the predictors for the LUR models for Windsor because they are evenly distributed across the entire city. Roadway density was significantly associated with eNO, whereas LUR estimates of individual traffic-related pollutants were not, suggesting that local roadways may be a significant source of exposure not captured by the LUR, or that roadway density may better represent the complex mixture of traffic-related air pollution, which includes combustion products, asphalt, and rubber. Reviews by Delfino (2002) and Sarnat and Holguin (2007) point out the difficulties in understanding which components of exhaust are the important pulmonary pathogens.

The present study contributes the following observations. First, in addition to major traffic arteries, local residential roadways may also adversely affect respiratory health. Second, traffic-related pollution may induce airway inflammation as indicated by increased concentrations of eNO. Third, eNO may be a more sensitive measure of air pollution toxicity than spirometry, suggesting that inflammation may precede airway narrowing. Finally, local roadway density may be a better indicator of traffic-related airway inflammation than LUR estimates of individual air pollutants. It is possible that the LUR model for Windsor had insufficient small-scale spatial variability, or that the roadway density provides a better estimate of the complex mix of traffic-related emissions than any single measured air pollutant.

## Figures and Tables

**Table 1:** Characteristics of Windsor elementary schoolchildren by indicator of air pollution exposure (mean ± SD; *n* = 2,328).

			Indicator of exposure to air pollution			
Characteristic	No.	NO_2_ (ppb)	SO_2_ (ppb)	PM_2.5_ (μg/m^3^)	Length of local roadways within 200 m of the home (km)	Length of all roadways within 200 m of the home (km)
Sex						
Male	1,164	13.53 ± 2.68	5.37 ± 0.75	15.61 ± 0.92	1.53 ± 0.59	1.67 ± 0.59
Female	1,190	13.62 ± 2.61	5.41 ± 0.74	15.63 ± 0.91	1.56 ± 0.56	1.70 ± 0.57
Race						
Caucasian	1,740	13.36 ± 2.50	5.32 ± 0.68	15.64 ± 0.91	1.58 ± 0.55	1.71 ± 0.56
Other	614	14.19 ± 2.93	5.58 ± 0.87	15.57 ± 0.95	1.46 ± 0.63	1.60 ± 0.64
Any smoker at home						
Yes	540	14.29 ± 2.73	5.66 ± 0.85	15.67 ± 0.83	1.63 ± 0.49	1.78 ± 0.49
No	1,814	13.36 ± 2.58	5.31 ± 0.69	15.61 ± 0.94	1.52 ± 0.59	1.65 ± 0.60
Any pets at home						
Yes	1,245	13.46 ± 2.53	5.36 ± 0.70	15.63 ± 0.89	0.54 ± 1.75	0.54 ± 1.85
No	1,109	13.71 ± 2.77	5.43 ± 0.80	15.61 ± 0.95	0.60 ± 1.65	0.62 ± 1.75
Cold/bronchitis/pneumonia in preceding 2 weeks						
Yes	327	13.68 ± 2.67	5.43 ± 0.75	15.55 ± 0.86	1.54 ± 0.58	1.67 ± 0.59
No	2,027	13.5 ± 2.64	5.38 ± 0.75	15.63 ± 0.93	1.55 ± 0.57	1.68 ± 0.58
Any medication for wheezing/asthma in preceding 2 weeks						
Yes	187	13.63 ± 2.55	5.45 ± 0.75	15.59 ± 0.83	1.57 ± 0.56	1.69 ± 0.55
No	2,027	13.57 ± 2.65	5.38 ± 0.75	15.62 ± 0.92	1.54 ± 0.57	1.68 ± 0.58

**Table 2:** Annual average concentrations of air pollutants estimated by LUR and roadway density around the home, resolved to the level of the postal code.

Exposure metric	Mean	5th percentile	Median	95th percentile	Interquartile range
Pollutant					
NO_2_ (ppb)	13.58	9.92	13.15	18.01	4.50
SO_2_ (ppb)	5.39	4.38	5.28	6.92	0.94
Coarse PM (μg/m^3^)	7.25	6.02	7.27	8.23	0.77
PM_2.5_ (μg/m^3^)	15.62	14.17	15.42	17.17	1.23
Black smoke (10^−5^/m)	0.75	0.61	0.75	0.87	0.11
Roadways (km)					
Distance to bridge	7.25	1.72	7.41	12.67	4.77
Length of local roadways within 200 m of the home	1.55	0.20	1.70	2.20	0.65
Length of all roadways within 200 m of the home	1.68	0.20	1.80	2.35	0.65

**Table 3:** Observed FEV_1_, FVC, and eNO by tertile of indicator of air pollution exposure.

					eNO (ppb)			
Exposure metric	No.^a^	Tertiles of air pollution	FEV_1_ [L (mean ± SE)]	FVC [L (mean ± SE)]	No.^b^	Mean ± SE	Median	Geometric mean
Air pollutant								
NO_2_ (ppb)	770	< 12.12	2.19 ± 0.01	2.53 ± 0.02	533	16.66 ± 0.79	09.92	11.6213
	767	12.12–14.44	2.17 ± 0.01	2.51 ± 0.02	559	15.23 ± 0.60	10.44	11.5182
	791	> 14.44	2.15 ± 0.01	2.49 ± 0.02	521	16.91 ± 0.77	10.85	12.0713
SO_2_ (ppb)	713	< 4.99	2.18 ± 0.01	2.52 ± 0.02	532	17.11 ± 0.81	10.16	11.9157
	767	4.99–5.49	2.18 ± 0.01	2.53 ± 0.02	557	14.92 ± 0.58	10.54	11.4138
	788	> 5.49	2.14 ± 0.01	2.48 ± 0.02	524	16.77 ± 0.63	10.40	11.8766
Coarse PM (μg/m^3^)	769	< 7.04	2.18 ± 0.01	2.52 ± 0.02	569	15.48 ± 0.63	10.17	11.5898
	769	7.04–7.53	2.19 ± 0.02	2.53 ± 0.02	528	16.73 ± 0.76	10.56	11.7705
	790	> 7.53	2.14 ± 0.01	2.48 ± 0.02	516	16.59 ± 0.79	10.29	11.8366
PM_2.5_ (μg/m^3^)	828	< 15.19	2.16 ± 0.01	2.51 ± 0.02	575	16.08 ± 0.70	10.33	11.5169
	706	15.19–15.96	2.17 ± 0.02	2.50 ± 0.02	480	15.80 ± 0.76	09.91	11.3482
	794	> 15.96	2.18 ± 0.01	2.52 ± 0.02	558	16.79 ± 0.72	10.74	12.2922
Black smoke (10^−5^/m)	769	< 0.72	2.18 ± 0.01	2.52 ± 0.02	477	15.34 ± 0.70	9.83	11.1738
	769	0.72–2.78	2.18 ± 0.01	2.53 ± 0.02	558	17.12 ± 0.74	11.10	12.3770
	790	> 2.78	2.15 ± 0.01	2.49 ± 0.02	578	16.45 ± 0.73	10.50	11.7795
Roadways (km)								
Distance to bridge	767	< 5.78	2.16 ± 0.01	2.50 ± 0.02	511	17.04 ± 0.78	12.1920	10.91
	769	5.78–8.72	2.15 ± 0.01	2.49 ± 0.02	558	15.11 ± 0.60	11.3575	10.18
	792	> 8.72	2.18 ± 0.01	2.53 ± 0.02	544	16.54 ± 0.76	11.6586	09.94
Length of local roadways within 200 m of the home	793	< 1.45	2.18 ± 0.01	2.52 ± 0.02	558	16.18 ± 0.76	11.4346	09.88
	762	1.45–1.90	2.15 ± 0.02	2.50 ± 0.02	514	16.29 ± 0.72	11.7836	10.72
	773	> 1.90	2.17 ± 0.01	2.51 ± 0.02	541	16.14 ± 0.65	11.9506	10.68
Length of all roadways within 200 m of the home	816	< 1.60	2.17 ± 0.01	2.52 ± 0.02	565	15.71 ± 0.74	11.1329	09.69
	824	1.60–2.00	2.17 ± 0.01	2.50 ± 0.02	566	16.22 ± 0.65	11.9555	10.67
	688	> 2.00	2.16 ± 0.02	2.51 ± 0.02	482	16.76 ± 0.76	12.1588	10.85

a *n* = 2,328 for FEV_1_ and FVC.

b *n* = 1,613 for eNO.

**Table 4:** Adjusted associations between indicators of exposure to air pollution and pulmonary physiologic measures [β (95% CI)].^a^

Exposure metric	Percent predicted FEV_1_	Percent predicted FVC	Ln(eNO) (× 10^3^)
Pollutant			
NO_2_ (ppb)	0.03 (−0.14 to 0.21)	0.10 (−0.09 to 0.28)	3.9 (−10.8 to 18.7)
SO_2_ (ppb)	−1.09 (−3.32 to 1.14)	−0.31 (−1.43 to 0.81)	0.6 (−54.6 to 54.8)
Coarse PM (μg/m^3^)	0.04 (−0.65 to 0.73)	0.17 (−1.25 to 1.60)	0.02 (−0.03 to 0.08)
PM_2.5_ (μg/m^3^)	−0.21 (−1.84 to 1.41)	−0.74 (−2.47 to 0.98)	38.7 (−1.07 to 78.4)*
Black smoke (10^−5^/m)	−1.46 (−17.38 to 14.43)	3.29 (−2.13 to 8.72)	200.6 (−222.3 to 623.4)
Roadways (km)			
Distance to bridge	0.006 (−0.13 to 0.14)	−0.02 (−0.16 to 0.13)	−3.2 (−14.9 to 0.86)
Length of local roadways within 200 m of the home	−0.15 (−1.27 to 0.96)	−0.70 (−1.88 to 0.48)	65.6 (1.5 to 129.8)**
Length of all roadways within 200 m of the home	−0.01 (−1.08 to 1.05)	−0.47 (1.60 to 0.66)	96.4^b^ (34.2 to 158.7)#

a For FEV_1_ and FVC, *n* = 2,328; for eNO, *n* = 1,613.

b This is equivalent to a 10.1% (95% CI, 3.5 to 17.2) increase in eNO for a 1-km increase in roadways.

* *p* < 0.10.

** *p* < 0.05.

# *p* < 0.01.

**Table 5:** The association between roadway density and eNO and fine PM air pollution, stratified by history of physician-diagnosed asthma.

	Physician-diagnosed asthma [β^a^ (95% CI)]	
Exposure metric	Yes (*n* = 330)	No (*n* = 1,283)
PM_2.5_ (μg/m^3^)	76.40 (−45.84 to 198.65)	13.55 (−35.70 to 62.80)
	*p* = 0.2193	*p* = 0.5894
Length of all roadways within 200 m of the home (km)	256.15^b^ (52.52 to 459.78)	77.60 (0.51 to 154.68)
	*p* = 0.0139	*p* = 0.0485

a β is the regression coefficient for the natural logarithm of eNO multiplied by 1,000.

b This is equivalent to a 29.2% (95% CI, 5.4 to 58.4) increase in eNO for a 1-km increase in roadway.
